# Machine learning based canine posture estimation using inertial data

**DOI:** 10.1371/journal.pone.0286311

**Published:** 2023-06-21

**Authors:** Marinara Marcato, Salvatore Tedesco, Conor O’Mahony, Brendan O’Flynn, Paul Galvin

**Affiliations:** Tyndall National Institute, University College Cork, Cork, Ireland; Menoufia University, EGYPT

## Abstract

The aim of this study was to design a new canine posture estimation system specifically for working dogs. The system was composed of Inertial Measurement Units (IMUs) that are commercially available, and a supervised learning algorithm which was developed for different behaviours. Three IMUs, each containing a 3-axis accelerometer, gyroscope, and magnetometer, were attached to the dogs’ chest, back, and neck. To build and test the model, data were collected during a video-recorded behaviour test where the trainee assistance dogs performed static postures (standing, sitting, lying down) and dynamic activities (walking, body shake). Advanced feature extraction techniques were employed for the first time in this field, including statistical, temporal, and spectral methods. The most important features for posture prediction were chosen using Select K Best with ANOVA F-value. The individual contributions of each IMU, sensor, and feature type were analysed using Select K Best scores and Random Forest feature importance. Results showed that the back and chest IMUs were more important than the neck IMU, and the accelerometers were more important than the gyroscopes. The addition of IMUs to the chest and back of dog harnesses is recommended to improve performance. Additionally, statistical and temporal feature domains were more important than spectral feature domains. Three novel cascade arrangements of Random Forest and Isolation Forest were fitted to the dataset. The best classifier achieved an f1-macro of 0.83 and an f1-weighted of 0.90 for the prediction of the five postures, demonstrating a better performance than previous studies. These results were attributed to the data collection methodology (number of subjects and observations, multiple IMUs, use of common working dog breeds) and novel machine learning techniques (advanced feature extraction, feature selection and modelling arrangements) employed. The dataset and code used are publicly available on Mendeley Data and GitHub, respectively.

## 1 Introduction

Animals express their feelings and emotions through behaviour, therefore behavioural monitoring offers the opportunity to deepen our understanding of animal health and well-being. Ethogram markings have been traditionally used to record and qualify a set of behaviours shown in a particular setting for a determined duration, allowing the discovery of patterns, similarities and differences between subjects [1, 2]. Accordingly, researchers and experts typically use ethograms to characterise, quantify and monitor animal behaviour in order to evaluate well-being and temperament [3].

Ethograms are usually designed for each experimental protocol to include relevant behaviours considering the subjects and research questions under investigation. Their implementation normally involves manual annotation performed by experts during the observation session, or afterwards through video analysis of recorded sessions [1]. This method is time-consuming and may require expert knowledge, therefore ethogram markings are generally employed in short sessions with a selected group of subjects.

In order to overcome the challenges related to obtaining individual-level behavioural data on dogs in the medium and long term, smart canine activity monitors have been developed. The recent evolution of motion sensing, processing power and artificial intelligence technology has enabled the development of automated systems for behavioural estimation and monitoring [4–11]. They not only eliminate the need for direct observation and assessment by trained professionals but also remove the subjectivity and error associated with human markings. Moreover, they allow continuous recording with an unprecedented level of detail.

Machine learning techniques, such as supervised learning algorithms, have been employed to derive a classification model based on the data provided by inertial measurement units (IMUs) [5–9, 12]. The units are usually composed of a 3-axis accelerometer, gyroscope, and/or magnetometer, providing a reliable, accurate, cost-effective, and energy-efficient solution for motion analysis. Moreover, these sensors are small, lightweight, and low-cost. These have significant advantages when compared to motion recognition systems based on image analysis from camera systems [13–16] which pose constraints in terms of mobility relative to the camera and algorithm complexity.

The aim of this study was to develop the first posture estimation system specifically for working dogs to create automatic ethograms considering five canine behaviours (walking, standing, sitting, lying down, and body shake). The data collection was designed to consider the specific application of this system to working dogs. Accordingly, only two common working dog breeds were included and multiple IMUs were positioned to emulate the harness used by dogs.

The data preprocessing technique utilised advanced feature extraction methods for the first time in this field. Three novel machine learning architectures were implemented and evaluated to improve the rate of correct detection of less frequent behaviour. The analysis of feature importance indicated that the back was the most influential sensor position and accelerometers were the most critical sensor type for posture estimation. The classification results achieved in this study demonstrate the superior performance of this model compared to previous research.

The potential applications of canine activity monitoring in the working dog industry which motivated the development of this work are presented in Section 1, while Section 2 examines the state-of-the-art in this area and identifies the research gaps. Finally, Section 3 elaborates on the novelty and highlights of this study.

### 1.1 Motivation

The COVID-19 pandemic has profoundly changed people’s behaviour [17], and impacted dog-owner affective experiences and relationships [18], which in turn, correlate with dog’s physical activity [19]. Smart canine activity monitors have been developed to accurately and reliably monitor activity and health parameters to indicate overall well-being [9, 20, 21]. In order to achieve that, they need to first quantify activity levels and identify important behaviours. Subsequently, such data could be fed to specific algorithms to extract meaningful information about the targeted application.

This technological advancement provides an enormous opportunity not only for pet owners but also for working dog organisations including those involved in guide, assistance, police, search and rescue dogs, etc. These devices could be used on guide dogs to provide their organisation with well-being information while they are off-site working with their visually-impaired partners, as a means to address the current challenges in assessing and reporting well-being. They could also enable communication between assistance dogs and their partner’s support systems, such as carers and healthcare professionals, as these dogs are trained to perform specific postures and behaviours to assist their partners. This also applies to search and rescue dogs, who are trained to communicate with their handlers through body postures while working remotely [4, 22, 23], and open-field guard or shepherd dogs [7]. In the case of all types of working dogs, they could also assist in creating automatic scoring for the assessment of behavioural performance [24] and computer-canine training system [10, 25, 26]. Activities estimated by a posture recognition algorithm during a behaviour test could be used to predict ethogram items, rater scoring or ultimately the dog’s probability of success in their training programme [24].

This technology enables a range of applications including assessment of well-being and injury recovery. Canine activity monitors could be used for home activity monitoring of pet dogs with chronic conditions especially those affected by orthopaedic, neuromuscular, or neurological illnesses such as osteoarthritis, muscular dystrophy [27] or heart failure [28]. For example, they could provide an objective assessment method of evaluating dogs during an injury recovery process [9] and suffering from osteoarthritis [29, 30]. They could also be used to monitor skin conditions by identifying pruritic behaviours in allergic dogs and the occurrence of seizures in epileptic dogs [31].

Another possible application of canine posture recognition systems regards the monitoring of well-being as some activity and health parameters have been associated with the occurrence of emotional states and stress in dogs [20, 32]. They could also be utilised for noise cancellation in the interpretation of physiological measurements [4, 33]. Furthermore, they could also be used to measure activity at night, as rest was positively associated with success in apprentice guide dogs [34], and static and inactive behaviours were associated with anxiety and hyper-vigilance [35].

### 1.2 Related work

The work presented in this manuscript builds upon the existing literature on posture recognition systems for dogs. These systems can be classified according to the type of hardware and software technology deployed. The former relates to the method for gathering data for posture classification including inertial (accelerometer and gyroscope) or image (camera) based systems [36]. The latter is responsible for estimating posture based on data from sensors. It can take place in either real-time or non-real-time and be embedded, computer, or cloud-based.

Several classification algorithms featuring statistical models [10], supervised machine learning [5, 6, 9] or knowledge engineering [11] techniques have been deployed to identify key canine postures and activities. In particular, this work features an inertial sensing, non-real-time, computer-based posture classification system.

The possibility of estimating canine posture using IMUs was first investigated by Ribeiro et al. [22] and Brugarolas et al. [25], and subsequent attempts were introduced later [5, 6, 11]. Since then the design of several posture recognition systems has been reported in the literature using both custom-designed [4, 5, 25] and commercial inertial systems [8, 9, 12, 37, 38]. Some canine activity monitoring devices are currently commercially available [21, 35, 39, 40].

Table 1 summarises, in chronological order, nine published studies whose main goal was to develop or evaluate a canine posture estimation system based on inertial sensors. It includes information on subjects, sensors and placement, data collection methods, data pre-processing techniques, classification algorithms, as well as train-test split and results.

**Table 1:** Canine posture recognition algorithms using IMUs reported in literature.

Publication	Subjects Sensors & Placement	Postures Data Collection	Pre-processing Classification Algorithm	Train-Test Split / Results
[11]	5 Urban Search and Rescue dogs (breed not available) Custom-designed system 2-axis ACC Wither and rump	3 static: standing, sitting, lying down 1 dynamic: walking Postures repeated 5 times	Window size of 2 s and overlap is not reported Calculation of angles from ACCs Algorithm: custom, uses angles of ACCs	Training and test sets contain the same dogs from [22] Overall accuracy was 80%
[9]	18 dogs (13 breeds) AX3, Axivity ACC @ 30Hz Neck collar	17 behaviours: bark, chew, dig, drink, eat, excrete, jump, lie down, sit, pawing, run, shake, shiver, sniff, urinate, walk, unspecified Sessions lasted 20-40 minutes, freely moving dogs	Window size of 1 s and overlap of 0.5 s PCA-based feature extraction (50 components, 95% of variance) Normalisation using ECDF Algorithm: K-nearest Neighbours (KNN) with k = 1	10-fold stratified cross-validation Overlap between training and test frames can be 48% Overall accuracy was 69%
[7]	24 dogs (12 BM, 12 LR) Custom-designed system ACC and GYR @ 100Hz Back	3 static: sit, lay, stand 4 dynamic: run, trot, walk, bark, search Postures recorded on 2 days, sessions lasted 10min	Window size of 1 s and overlap is not reported 126 Features: 69 from GYR, 45 from ACC Algorithm: Support Vector Machine (SVM) Hyperparameter optimisation: soft margin (C) and gamma	5-fold cross-validation, controlling for subjects Single dog accuracy: Intra-subject (BM 91%, LR 92%) Intra-breed Inter-subject (BM 70%, LR 73%) Inter-breed (74%) Multiple dog accuracy: Inter-subject 83%
[5]	7 dogs (6 LR, 1 KK) Custom-designed system ACC and GYR @ 10Hz Rump, Chest, Abdomen, and Withers	5 static: sitting, standing, lying down, eating off ground, standing on two legs 3 dynamic: walking, climb stairs, down ramp Static postures repeated 5 times for 4s + climb stairs, down ramp	Window size of 1 s and overlap is not reported Moving average filter Cascade machine learning algorithm C1. separate static behaviour and classify dynamic behaviour C2. posture classification for static behaviour	5-fold cross validation The test set was 30% of the data Intra-subject accuracy: C1. 92-100% C2. 78-99.5% Inter-subject accuracy: C1. 0-100% C2. NA
[6]	2 dogs (1 LR, 1 KK) Custom-designed system ACC and GYR @ 10 Hz Neck and rump	5 static: sit, stand, lie, eat off the ground, stand on two legs Static postures repeated 5 times for 4s on 2 days	Window size and overlap are not reported Moving average filter Algorithm: Random Forest (RF), KNN, and Logistic Model Tree (LMT) Two-stage cascade learning technique used: C1. separated postures and transitions (ACC, GYR) C2. classification of 5 postures (ACC)	10-fold cross validation Inter-subject accuracy: C1. 81—92% C2. 92 -100%
[12]	2 dogs (Pembroke Welsh Corgi, Toy Poodle) AX3, Axivity ACC @ 25Hz Neck Collar	10 behaviours: walking, eating, sitting, laying, sniffing, running, jumping, drinking, shaking, scratching No information available on data collection procedures	Window size of 1 s and overlap 0.96 s Features from PCA and ECDF Algorithm: K-nearest Neighbours (k = 1) Distance metric: Euclidean Distance (ED), Dynamic Time Warping (DTW), DTW-D proposed by [41]	Training and test sets contain the same dogs Distance metric DTW-D outperformed ED and DTW Mean F1-measure 67% and accuracy is 78%
[8]	24 dogs (20 breeds) GT9X Link, ActiGraph ACC and GYR @ 100Hz Neck collar	7 behaviours: stand, sit, lie down, walk, trot, gallop, sniff Each task lasted 3 minutes	Window size of 2 s and overlap is not reported 27 features created and Forward Feature Selection (FFS) Algorithm: Linear and Quadratic Discriminant Analysis classifier (LDA and QDA)	10-fold cross validation Train-test split criteria missing Accuracies All: (27 features) LDA 74%, QDA 73% FFS: (8 feat) LDA 73%, (5 feat) QDA 76%
[37]	21 dogs (19 breeds) Apple Watch Series 1 ACC and GYR @ 50 Hz Neck collar	8 behaviours: sit, lay, stand, walk, trot, run, eat, drink No information available on data collection procedures	Window size of 1.3 s and overlap of 0.8 s 252 features Models created for small, medium, and large-sized dogs (N = 7 in each group) Algorithm: SVM Hyperparameter optimisation: soft margin (C) and gamma	Leave one subject out Average accuracy 57% (results taken on comparable approach)
[38]	45 dogs (27 breeds) GT9X Link, ActiGraph ACC and GYR @ 100Hz Neck and back	3 static: sit, stand, lie down 4 dynamic: trotting, walk, play, treat-search Each task lasted 3 minutes	Window size of 2 s and overlap of 1s 54 features and FFS Algorithm: LDA, QDA, SVM, and Tree	Leave one subject out Accuracy using the best classifier (SVM): Back 91.4% and Neck 75.6%
This study	42 dogs (41 LR, 1 GR) GT9X Link, ActiGraph ACC and GYR @ 100Hz Back, Chest, and Neck	3 static: standing, sitting and lying down 2 dynamic: walking and body shake All postures for 1 minute, body shake spontaneously	Window size of 1 s and overlap of 50% Cascade machine learning architectures combining: Random Forests and Isolation Forests	Development set 10-fold group cross validation Test set Hold-out Inter-breed Inter-subject f1-weighted 0.90

Breeds: LR = Labrador Retriever, BM = Belgian Malinois, KK = Kai Ken. Sensors: ACC = Accelerometer, GYR = Gyroscope. All sensors are 3-axis except stated otherwise. Features: PCA = Principal Component Analysis, ECDF = empirical cumulative density function.

Even though most of the studies present overall accuracy as the main performance measure, comparisons across studies must be carefully made. This metric is highly influenced by class imbalance. In other words, accuracy becomes unrepresentative of the algorithm’s performance as the number of observations in each category becomes dissimilar. Moreover, using data from the same subjects or same breeds in the training and test sets causes the performance metric to be higher compared to using different subjects [5, 37] or breeds [7]. Controlling for these factors considering the final model’s intended application is vital in estimating its real-world performance. Several previously reported studies insufficiently reported the number of observations per class and subject, and the criteria used for splitting the original dataset into training and test sets, even though they significantly affect the model’s accuracy.

Some research reports failed to include information regarding important methodological details such as the device sampling rate [11], window size [6] and overlap [5, 7, 8, 11], data collection methods [12], and feature extraction methods [9]. Insufficient information and incompatible methodological designs pose great challenges when comparing the performance achieved in different studies and limit their contribution to state-of-the-art knowledge. Moreover, Kumpulainen et al. [38] was the only study to publicly share the dataset used for model development.

The number of subjects used for developing canine posture recognition algorithms was rather limited in early research; however, larger sample sizes were reported in more recent studies [7, 8, 37, 38]. Gerencser et al. [7] used two breeds of dogs and attained an inter-subject accuracy of 83%, and Kumpulainen et al. [8] used 20 breeds of dogs and achieved an accuracy of 76%. The lower figure in the latter study can be explained as the use of dogs from different breeds introduces more variability negatively impacting that metric. However, it is unclear whether the same subjects were used for training and testing the algorithm, which would positively affect that metric [7, 37]. To address these issues, this study included 41 dogs belonging to common working dog breeds.

Additionally, two studies were found whose aim was to evaluate commercially available canine activity monitors [35, 39]. These studies insufficiently describe the algorithm built as their primary objective was to assess and validate the classification model used in such commercially available devices, therefore they were not included in Table 1. The first one used PetDialog+ powered by Oggi (Tel Aviv, Israel) and Zoetis (Dublin, Ireland) smart collars on 51 dogs classifying 8 different activities including walking, trotting, canter/galloping, sleeping, static/inactive, eating, drinking, and head shaking. It achieved a mean balanced accuracy of 89% and f1-score of 95% [35].

The second study used Whistle smart collars (MacLean, USA) in a large study involving a canine posture training database containing data from over 2,500 dogs. The system was capable of identifying 14 activities, including nine behaviours: drinking, eating, licking object, licking self, petting, rubbing, scratching, and sniffing; and five postures: lying down, sitting, standing, vigorous, and walking. It achieved a mean balanced accuracy of 85% and macro f1-score of 69% [39] using group cross-fold validation.

Importantly, no study was found to develop a posture recognition system specifically for working dogs. This limitation in the state-of-the-art research was addressed in this research by collecting data on common working dog breeds and attaching multiple sensors to a harness similar to the one used by working dogs. None of the classification algorithms presented in the literature experimented with advanced feature extraction methods or employed anomaly detection classifiers to identify minority classes. Hence, these were explored in the present work which also addressed issues identified in previous research. In particular, it employed more robust cross-validation techniques and reported comprehensive performance metrics for a more complete evaluation of the model.

### 1.3 Novelty and contribution

The main goal of this work was to build a novel system capable of reliably identifying key canine behaviours in working dogs. In addition to the most common dog postures, namely: walking, sitting, standing, and lying down, it was also of interest to predict body shake as it characterises a coping behaviour shown when dogs experience acute stress [42]. This study comprises four main methodological novelties, advancing the state-of-the-art by:

1. providing the largest open access dataset for dog activity recognition specific to predominant working dog breeds, namely, Labrador Retrievers, Golden Retrievers, and their crosses [43–45];
2. applying advanced feature extraction methods for the first time in this field;
3. evaluating three novel architectures of machine learning classifiers to address the natural class imbalance, including anomaly detection;
4. utilising data from multiple IMUs (i.e., located by the neck, back, and chest) and investigating the effect of IMU placement in a large dataset.

With the advent of FAIR principles [46], a significant improvement has been observed as authors adhere to best practices by sharing raw datasets [37, 38, 47]. Accordingly, this work contributes to the state-of-the-art by providing the annotated dataset on Mendeley Data (link) [48]. The code developed to implement the methodology which yielded the results presented in this study was also made publicly available on GitHub (link). This work proposes a framework for future studies to address the methodological issues discussed in the detailed analysis of past studies. We attempt to establish a model to promote the interoperability of research outputs, considering the particular technicalities in the field of dog activity recognition.

## 2 Methods

### 2.1 Subjects

The subjects were 42 healthy apprentice dogs participating in the assistance dog programme at the Irish Guide Dogs for the Blind (IGDB)’s Training Centre in Cork, Ireland. The IGDB is a charitable organisation that provides guide and service dogs to help persons who are vision impaired and families of children with autism. The dogs were Golden Retrievers (N = 1, Mean Age = 14.6 months), Labrador Retrievers (N = 16, Mean Age = 17.3 month) and Crosses (N = 25, Mean Age = 11.6 months).

### 2.2 Ethical approval

The Health Products Regulatory Authority (HPRA) is the competent authority in Ireland responsible for the implementation of EU legislation (Directive 2010/63/EU) for the protection of animals used for scientific purposes. Practices not likely to cause pain, suffering, distress or lasting harm equivalent to, or higher than, that caused by the introduction of a needle in accordance with good veterinary practice fall outside the scope of HPRA Scientific Animal Protection Legislation. Consequently, no special permission from HPRA was required considering that the nature of the present work was non-invasive.

The Animal Ethics Experimentation Committee (AEEC) and Social Research Ethics Committee (SREC) at University College Cork (UCC) reviewed and approved this study’s data collection procedures as described below under request numbers 2019-007 and 2019-016, respectively. All participants were briefed on the experimental protocol and signed the written informed consent sheet.

### 2.3 Devices and data

Inertial data were gathered during the data collection session by three IMUs GT9X Links (Actigraph, Pensacola, USA) containing a three-axial accelerometer, gyroscope, and magnetometer. ActiLife software (Actigraph, Pensacola, USA) was used to initialise the devices with a 100Hz sampling rate.

**Fig 1:**
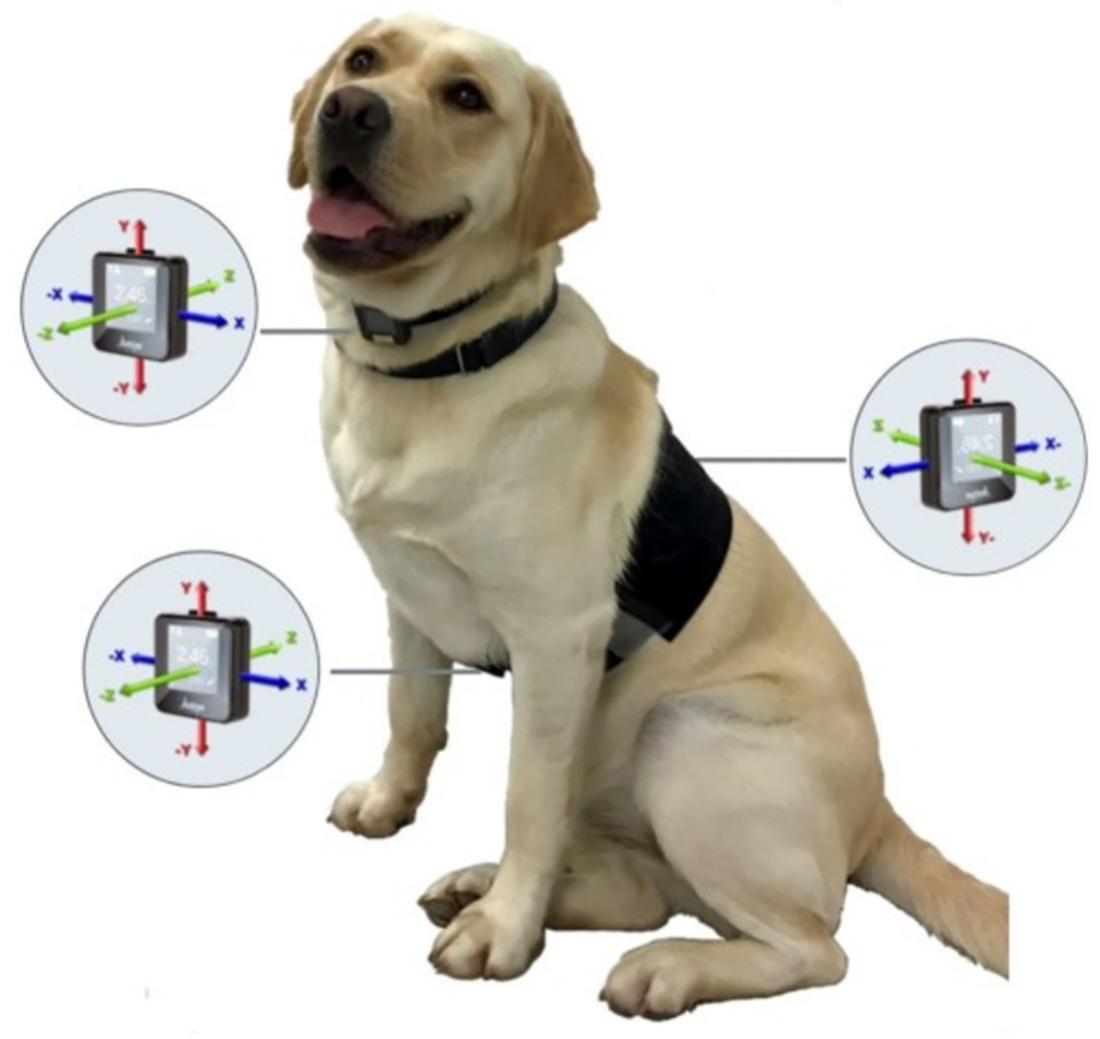
IMUs sensor placement on the dogs’ neck, back, and chest.

The IMUs were attached to fabric straps on the dogs through hook-and-loop fasteners glued to the devices and on the fabric straps (Xsens, Enschede, the Netherlands). The sensors were placed on the dogs’ neck, back, and chest following the placement shown in Fig 1.

The IMU Raw datasets were concatenated producing 27 data streams, as follows: 3 IMUs (neck, chest, and back) containing 3 sensors each (accelerometer, gyroscope, magnetometer) with 3 axes each (X, Y, Z). The magnetometer features were removed from the dataset as initial experiments confirmed that they were not as predictive of posture. Therefore, the resulting IMU Raw dataset comprised accelerometer and gyroscope measurements which contained a total of 18 columns.

### 2.4 Data collection protocol

Data collection sessions took place in a room with a test area of 11.5m x 8.5m at the IGDB Training Centre in Cork, Ireland. Firstly, the IMUs were initialised using the local time and placed on the dog to gather inertial data on canine postures. In order to synchronise the IMU data and video recording, the local time was shown at the start of the video recording. Then the behaviour test described in Table 2 was performed to gather information on the key activities.

**Table 2:** Behaviour Test Protocol for canine posture data monitoring and acquisition.

Sub-test	Description
Familiarisation	Handler walks in 2 steps, lets the dog off-lead, ignores the dog keeping arms closed for 1 min (wait).
Walking	Handler calls dog by name, praises dog, puts on lead, and walks the dog on a leash for 1 min.
Standing	Handler commands the dog to stand for about 1 min (stand/wait).
Sitting	Handler commands the dog to sit/wait for about 1 min (sit/wait).
Lying down	Handler commands the dog to lie down for about 1 min (down/stay).

Walking, standing, sitting, and lying down were performed following commands, while body shakes occurred spontaneously. Some subjects were more cooperative than others in following the instructions to perform and hold postures for the predetermined period of time. Hence, positive reinforcement methods were administered by the handler using rewards in the form of verbal praise and food to increase adherence to the protocol.

### 2.5 Data annotation

The postures performed by the dog in the video-recorded session were annotated and classified into two types, and five postures, as described in Table 3.

**Table 3:** Categories of behaviour analysed.

Type	Posture
Dynamic	Walking, Body shake.
Static	Standing, Sitting, Lying down.

Posture Timestamps datasets were created for each video-recorded data collection session second by second. They comprised of the timestamp for the start and finish video recording time for each posture described.

The IMU Raw dataset was combined with the Posture Timestamps dataset to form a unified annotated dataset entitled IMU Posture dataset which contains IMU Raw data, dog name and breed, data collection number, and the two labels, namely: posture and type. Transitions and miscellaneous body postures which did not fit any of the previous categories were manually excluded from the dataset. Table 4 shows the number of observations in the IMU Posture dataset per posture and type.

**Table 4:** Type, posture, and the number of observations in the IMU Posture dataset.

Type	Observations	Posture	Observations
Dynamic	475,802	Walking	466,502
		Body shake	9,300
Static	1,199,200	Standing	640,700
		Sitting	323,200
		Lying down	235,300
Total			1,675,002

### 2.6 Feature extraction

**Fig 2:**
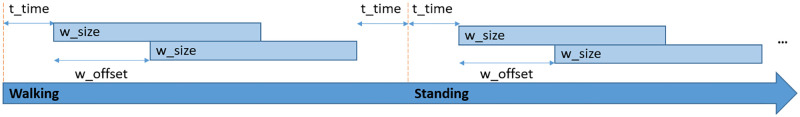
Rolling window used for selecting data for feature extraction. Hyper-parameters shown include re-sampling transition time (t_time) between postures, window size (w_size) used to calculate the statistical measures, and window offset (w_offset) to create separate observations.

Features were calculated using the IMU Posture dataset considering windows of time where a unique posture was performed. Three parameters control the creation of the window as illustrated in Fig 2 and described below:

- Transition time (t_time): time between different postures: t_time = 0.25*s*.
- Window size (w_size): rolling window size for calculating statistical measures: w_size = 1*s*.
- Window offset (w_offset): sampling offsets in the rolling window: w_offset = 0.5*s*.

The dataset was created by calculating diverse statistical measures on rolling windows controlled by the re-sampling hyper-parameters described above, following the standard 50% overlap between subsequent frames [9]. The feature set was obtained with tsfel package [49] which calculated 185 different variables for each of the 18 original raw inertial signals in the IMU Posture dataset, resulting in 3,330 features in the final set. These 185 features calculated by tsfel belonged to 60 types, including 26 spectral, 18 temporal, and 16 statistical features.

### 2.7 Classification models

All classification models were developed using Python (v3.8.1), in particular, tsfel (v0.1.4) was used to extract features, and sklearn (v1.0.2) and imblearn (v0.9.0) libraries were used to implement machine learning techniques. The models were trained on ICHEC’s Kay supercomputer nodes comprising of 2 x 20 core 2.4GHz Intel Xeon Gold 6148 processors [50].

The original dataset was split into three sets named development, golden, and test while controlling for the dogs in order to prevent overlap between datasets. The observation counts for each of these datasets after feature extraction using the rolling window are shown in Table 5. The development set contained 36 dogs and comprised 82% of the observations. The test set contained 5 dogs and consisted of 16% of the observations, and was created prioritising dogs who performed all the postures. The golden dataset contained data from the Golden Retriever dog only and represented the remaining 2% of the observations. The only dataset used for training models was the development set, the golden and test sets were used to analyse the intra-breed and inter-breed performance of the different models.

**Table 5:** Number of observations after feature extraction per dataset per posture.

Posture	Development	Test	Golden	Total
Body shake	82	18	2	102
Lying down	3,884	384	138	4,406
Sitting	5,096	946	156	6,198
Standing	9,286	2,158	588	12,032
Walking	6,418	1,360	398	8,176
Total	24,766	4,866	1,282	30,914

The development set was partitioned to create independent training and validation sets using 10-fold group cross-validation to ensure that those sets did not contain data from the same dogs. The resulting partitioned datasets were used to fit classification models in order to optimise the estimator hyper-parameters.

Select K Best method was utilised to select the best features by calculating ANOVA F-value between the features and labels. Scores given were grouped by sensor position and type to estimate their importance. Random Forest classifier was deployed to fit the development set using all features selected by Select K Best. Feature importance was grouped by the position and type of the sensor, and also by feature domain and type.

A grid search algorithm was utilised to evaluate all combinations of the hyper-parameters shown in Table 6. The grid search algorithm trained estimators individually on the relevant subset of the development set using each of the 1,000 hyper-parameter combinations. In particular, the feature selection algorithm was set to use the top K = 10, 20, 35, 55, and 80 features out of a total of 3,300 initial features extracted by tsfel. It also optimised two hyper-parameters of the Random Forest classifier, namely maximum depth = 3, 5, 7, and 10 and the number of estimators = 25, 50, 100, 250, and 500.

**Table 6:** Grid search hyper-parameter set for the classifiers.

Method	Hyper-parameters
Select K Best	k = [10, 20, 35, 55, 80]
Random Forest	max_depth = [3, 5, 7, 10]
	n_estimators = [25, 50, 100, 250, 500]

The performance metric chosen for optimising the estimators was f1-weighted, as it provides a good evaluation of the model’s ability to predict the correct class in multi-class classification problems with a natural class imbalance. Accordingly, the grid search algorithm chose the hyper-parameter combination used to create the estimators, which yielded the best f1-weighed score when fitted to the development set. Three novel classifiers were built using the optimal estimators selected by the grid search algorithm.

The next subsections outline different architectures used for combining the optimal estimators, in order to predict five postures, including three static postures (lying down, sitting, and standing) and two dynamic activities (walking and body shake). Classifier 1 comprised one estimator to predict the postures directly. Classifier 2 was a cascade classifier composed of 3 estimators, where the first one detected the type of posture (dynamic or static), and the other two further classified the posture. Classifier 3 had two estimators in sequence, where the first one detected body shakes as an anomaly, and the second one classified the remaining 4 postures.

#### 2.7.1 Classifier 1

**Fig 3:**
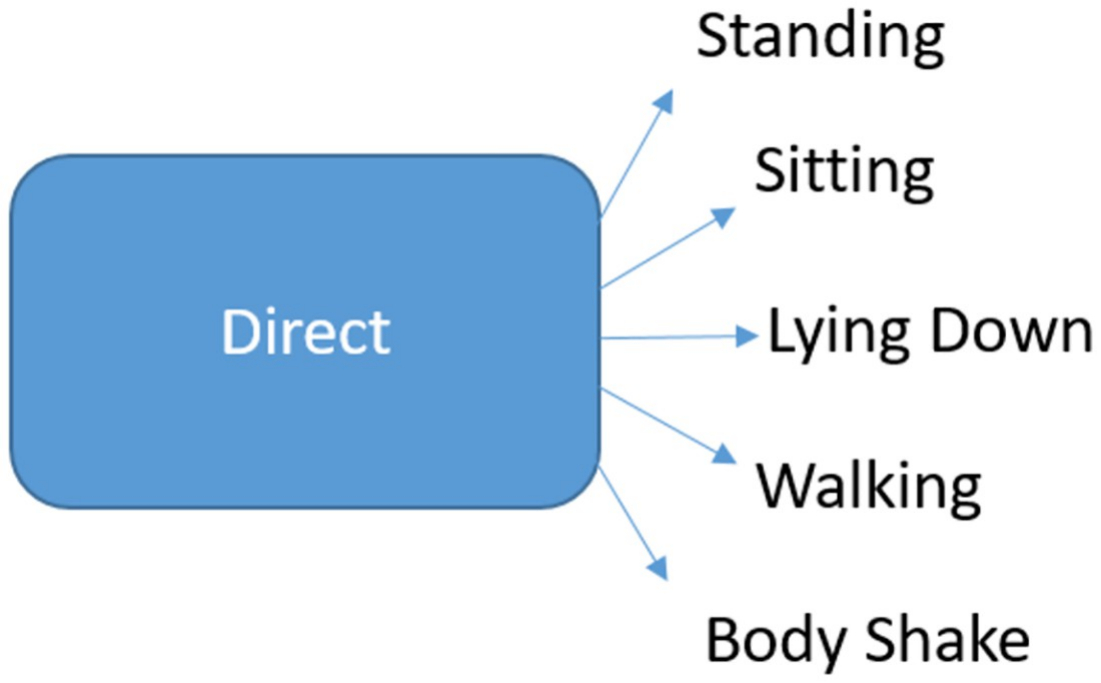
Direct estimator used in Classifier 1.

Random Forest classifier was fitted to the entire development dataset with all the 5 classes in order to predict posture directly as indicated in Fig 3.

#### 2.7.2 Classifier 2

**Fig 4:**
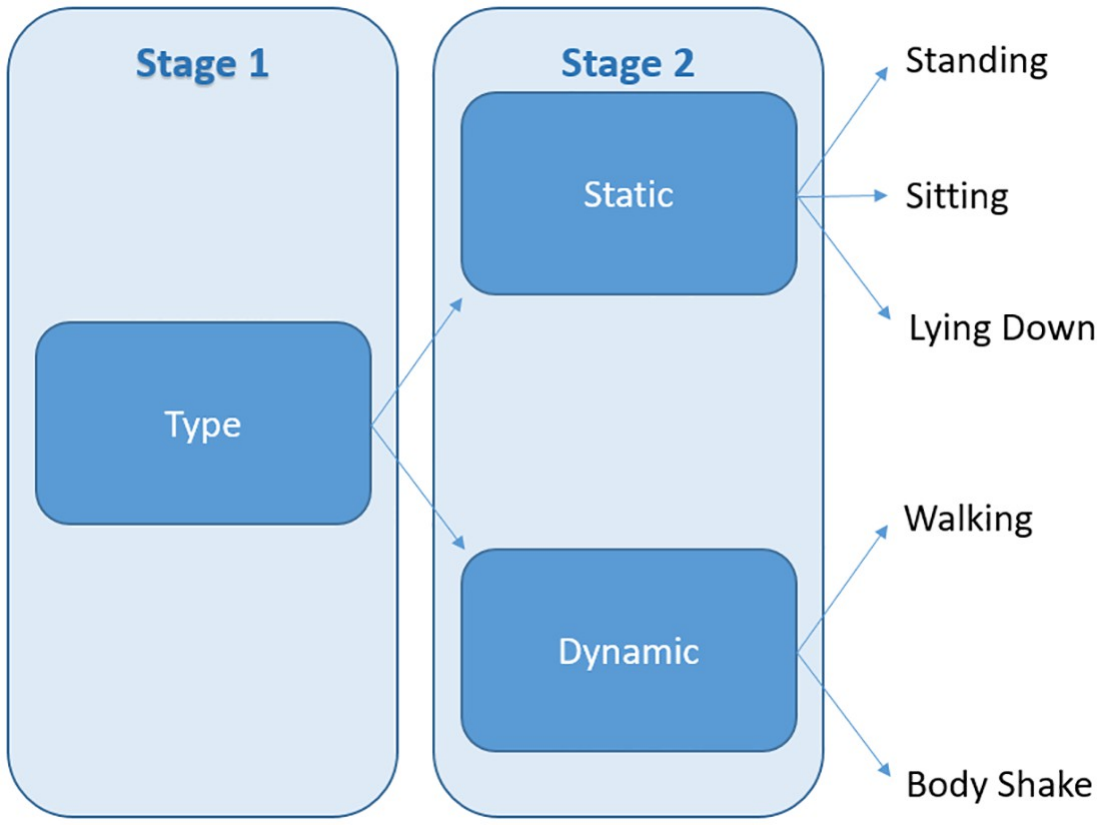
Type, static and dynamic estimators used in Classifier 2.

This classifier comprised three estimators, namely type, static and dynamic, as shown in Fig 4. Each estimator was trained separately, as outlined below:

- **Type**: Random Forest classifier was fitted to the entire development dataset to predict the label ‘type’ as described in Table 3 which comprised of two classes namely, static and dynamic.
- **Static**: Random Forest classifier was fitted to a subset of the dataset containing static postures to predict the classes lying down, sitting, and standing.
- **Dynamic**: Random Forest classifier was fitted to a subset of the dataset containing dynamic postures to predict the classes walking and body shake.

At prediction time, they were arranged sequentially in two stages to estimate the final posture as illustrated in the diagram in Fig 4. The class predicted by the type model in the first stage determined which estimator would be used in the second stage.

#### 2.7.3 Classifier 3

**Fig 5:**
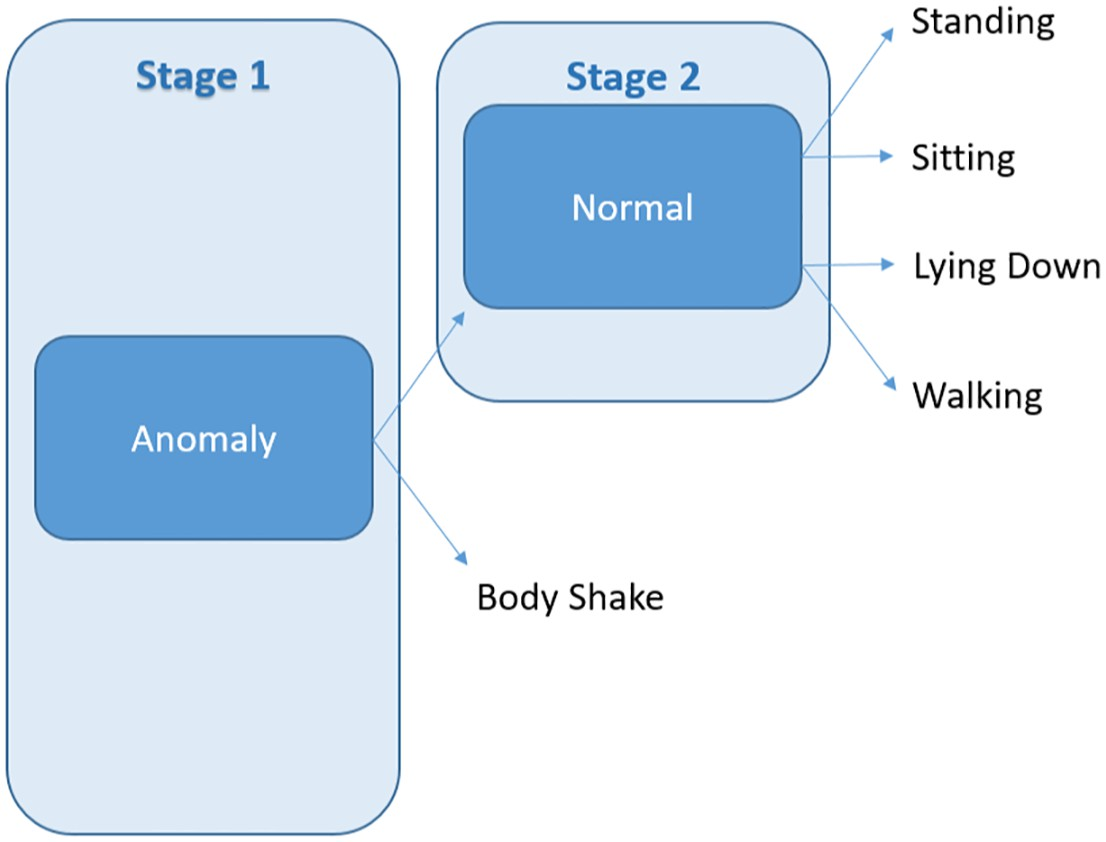
Anomaly and normal estimators used in Classifier 3.

This classifier comprised two estimators, namely anomaly and normal as indicated in Fig 5. In the first stage, it identified abnormal behaviour (body shake). Other instances went to the second stage where the normal estimator classified all the other four postures in the second stage.

- **Anomaly**: Isolation Forest, instead of Random Forest, was fitted to the entire dataset to identify the minority class body shake while all other four postures were assigned to the majority class. The hyper-parameter space searched for comprised of the following: number of estimators = 25, 50, 100, 250, 500, and contamination = 0.005, 0.01, 0.05, 0.1.
- **Normal**: Random Forest classifier was fitted to a subset of the dataset containing static postures to predict the classes lying down, sitting, standing, and walking.

### 2.8 Model performance

As discussed, a grid search algorithm was used to find the optimal hyper-parameter combination that would yield the model with the highest f1-weighted using 10-fold cross-validation. The optimal hyper-parameter set, and training and validation times were reported for the model that achieved the highest f1-weighted for each of the estimators composing the classifiers. The best estimators were built using the optimal hyper-parameter sets on the entire development set, and then used for predicting the label on unseen data. Intra-breed and inter-breed classification performance was evaluated using the test set (Labrador Retriever and Crosses), and the results are presented in the golden set (Golden Retriever only), respectively.

Besides the optimisation metric f1-weighted, other additional metrics were reported to facilitate the comparison between classifiers presented in this study and classifiers developed in other studies on canine posture recognition. In particular, the following metrics were reported for each of the postures: TPR (True Positive Rate, also known as Sensitivity or Recall), TNR (True Negative Rate, also known as Specificity), Accuracy, PPV (Positive Predictive Value, also known as precision), and f1 score. Hence, the following metrics were reported for each of the classifiers: f1-weighted, f1-macro, and confusion matrix. Confusion matrices were normalised considering the number of true instances in each class, i.e., the total count in the rows of the matrix.

## 3 Results

Section 3.1 shows the grid search’s chosen best model hyper-parameter combination, cross-validation results on the development set and performance on the test set evaluated individually and independently. Feature contribution was evaluated based on the f-classification score created by Select K Best and Random Forest classifier’s feature importance metric in Classifier 1. Section 3.2 presents the grouped figures as an estimate of the collective contribution of each sensor type and position.

The best models were deployed as indicated in the classifier diagram in Figs 3–5. They were evaluated on the test set to estimate intra-breed performance in Section 3.3, and the golden set to estimate inter-breed performance in Section 3.4. The performance achieved using the best classifier to predict posture on the combined test and golden sets is shown in Section 3.5.

### 3.1 Classification models

#### 3.1.1 Classifier 1

The best hyper-parameters chosen for the Direct estimator were K = 80 for Select K Best, and maximum depth = 10, and number of estimators = 250 for Random Forest. The time taken to build the estimator using the development set with 10-fold cross-validation was (in seconds): training time (M = 340.90, SD = 3.62) and validation time (M = 0.19, SD = 0.01). This model achieved an f1-weighted mean of 0.89 ±0.04 on the validation set, and an f1-weighted 0.89 and f1-macro 0.82 on the test set.

#### 3.1.2 Classifier 2

The best hyper-parameters chosen for the Type, Dynamic, and Static estimators are shown in Table 7 along with the training and validation times, and the f1-weighted performance metric for the validation and test sets.

**Table 7:** Classifier 2 model’s hyper-parameter set selected by grid search, the time (in seconds) taken to train and validate the model using the development set, and f1-weighted performance on 10-fold validation sets (mean ± standard deviation) and test set.

Model		Time		F1-weighted	
Estimator	Hyper-parameters	Training	Validation	Validation	Test
Type	k = 80 max_depth = 10 n_estimators = 100	138.17±1.47	0.12±0.00	0.95±0.02	0.95
Dynamic	k = 10 max_depth = 10 n_estimators = 25	1.38±0.05	0.03±0.00	0.98±0.01	0.99
Static	k = 80 max_depth = 10 n_estimators = 500	471.11±7.92	0.20±0.00	0.93±0.03	0.93

#### 3.1.3 Classifier 3

The best hyper-parameters chosen by grid search for the Anomaly and Normal estimators are shown in Table 8 along with the training and validation times, and the f1-weighted performance metric for the validation and test set.

**Table 8:** Classifier 3 model’s hyper-parameter set selected by grid search, and f1-weighted performance on 10-fold validation sets (mean ± standard deviation) and test set.

Model		Time		F1-weighted	
Estimator	Hyper-parameters	Training	Validation	Validation	Test
Anomaly	k = 10 n_estimators = 50 contamination = 0.005	4.63±0.09	0.18±0.01	0.99±0.03	0.95
Normal	k = 80 max_depth = 10 n_estimators = 500	685.07±11.61	0.27±0.01	0.90±0.03	0.90

### 3.2 Feature importance

Select K Best used f-classification to calculate the f-score and p-value for each feature taking the posture label into account. The score given was then normalised and grouped by sensor position (back, chest, and neck) and sensor type (accelerometer and gyroscope) to estimate their collective contribution to the model. These results are shown in Table 9 and indicate that the most important sensor position is on the back and the most important sensor type is the accelerometer for distinguishing between different postures.

**Table 9:** Feature scores calculated by f-classification in Select K Best by IMU position and sensor.

Position	SKB Score	Type	SKB Score
Back	0.45	Acc	0.26
		Gyr	0.19
Chest	0.39	Acc	0.22
		Gyr	0.17
Neck	0.16	Acc	0.09
		Gyr	0.07

Table 10 shows the feature importance given by the Random Forest from Classifier 1 on the 80 features chosen by Select K Best. No features derived from the neck sensor were among the 80 highest-ranked ones. Once again, these results indicate that the most important sensor position was on the back and the most important sensor type was the accelerometer.

**Table 10:** Feature importance calculated by Random Forest classifier considering the 80 features previously selected by Select K Best.

Position	RF Importance	Type	RF Importance
Back	0.57	Acc	0.37
		Gyr	0.20
Chest	0.43	Acc	0.24
		Gyr	0.19

Table 11 shows more information on the 80 highest-scoring features chosen by Select K best including their domain and collective importance. They belonged to 26 feature types out of the 60 domains calculated, specifically, there were 13 out of 26 spectral, 7 out of 18 temporal, and 5 out of 16 statistical features. In order of importance, the domains providing the best features were the statistical, temporal, and spectral domains. Even though the spectral domain had the biggest number of statistical feature types selected, it provided the least collective importance as calculated by Random Forest.

**Table 11:** Feature importance calculated by Random Forest classifier considering the 80 features selected by Select K Best by domain.

Domain (N)	Importance	Feature Type
Statistical (8)	0.47	Root mean square, median absolute deviation, empirical cumulative distribution function percentile, interquartile range, minimum, standard deviation, histogram, mean absolute deviation
Temporal (5)	0.32	Area under the curve, peak to peak distance, negative turning points, entropy, positive turning points
Spectral (13)	0.21	wavelet absolute mean, wavelet standard deviation, spectral distance, wavelet energy, linear prediction cepstral coefficients, spectral skewness, spectral decrease, spectral centroid, spectral slope, spectral kurtosis, maximum frequency, wavelet variance, spectral roll-off

### 3.3 Intra-breed evaluation

A summary of the classification metrics on the test set for Classifiers 1, 2, and 3 per class, and f1-weighted and f1-macro averages is shown in Tables 12–14, respectively. The three classifiers achieved very similar results. Classifier 3 achieved the best f1-macro as a result of its improved ability to correctly identify body shakes; however, its f1-weighted was slightly lower for standing and lying down.

**Table 12:** Classification metrics per posture achieved using the best models selected by grid search in Classifier 1 on the test set.

Posture	TPR	TNR	Accuracy	PPV	F1-score
Body shake	0.44	1.00	1.00	0.62	0.52
Lying down	0.93	0.99	0.98	0.88	0.90
Sitting	0.91	0.97	0.96	0.87	0.89
Standing	0.87	0.94	0.91	0.92	0.89
Walking	0.92	0.95	0.94	0.88	0.90
f1-weighted	0.89				
f1-macro	0.82				

**Table 13:** Classification metrics per posture achieved using the best models selected by grid search in Classifier 2 on the test set.

Posture	TPR	TNR	Accuracy	PPV	F1-score
Body shake	0.39	1.00	1.00	0.54	0.45
Lying down	0.95	0.99	0.99	0.88	0.91
Sitting	0.91	0.97	0.96	0.87	0.89
Standing	0.87	0.94	0.91	0.92	0.90
Walking	0.92	0.95	0.94	0.88	0.90
f1-weighted	0.90				
f1-macro	0.81				

**Table 14:** Classification metrics per posture achieved using the best models selected by grid search in Classifier 3 on the test set.

Posture	TPR	TNR	Accuracy	PPV	F1-score
Body shake	0.61	1.00	1.00	0.50	0.55
Lying down	0.93	0.99	0.98	0.87	0.90
Sitting	0.91	0.97	0.96	0.87	0.89
Standing	0.87	0.94	0.91	0.92	0.89
Walking	0.91	0.96	0.94	0.89	0.90
f1-weighted	0.89				
f1-macro	0.83				

**Fig 6:**
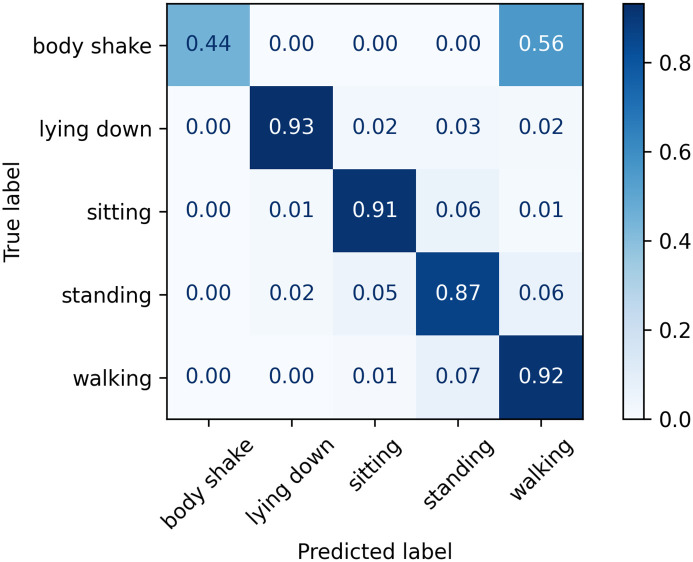
Confusion matrix for Classifier 1 on the test set.

**Fig 7:**
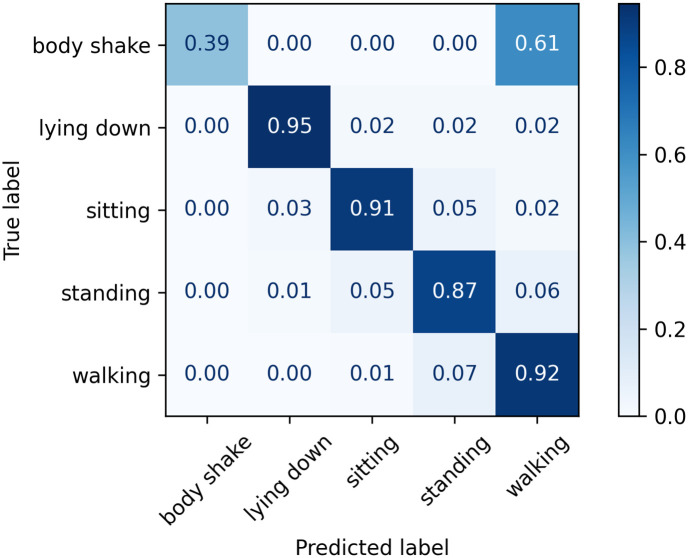
Confusion matrix for Classifier 2 on the test set.

**Fig 8:**
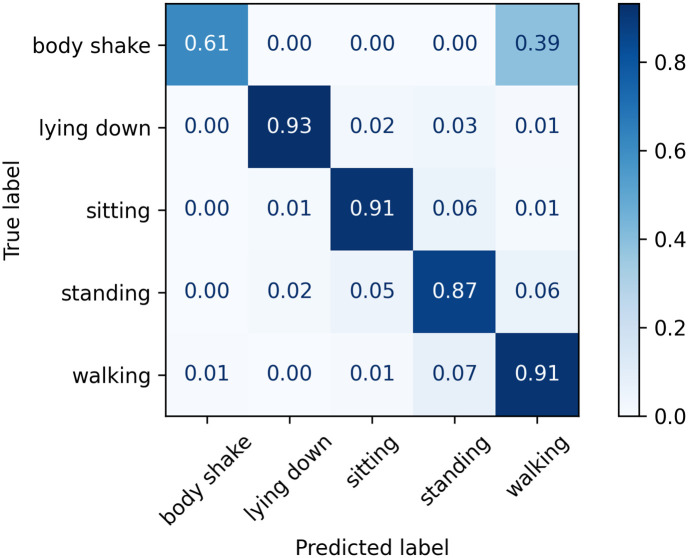
Confusion matrix for Classifier 3 on the test set.

The normalised confusion matrices with the predicted labels on the test set using Classifier 1, 2, and 3 models are shown in Figs 6–8, respectively. The main difference appears in the body shake performance as this class was mainly correctly classified by Classifier 3 model only. This improved performance in the minority class did not negatively affect the model’s ability to identify other classes as they showed comparable figures to Classifiers 1 and 2.

### 3.4 Inter-breed evaluation

The golden dataset was used to evaluate the performance of each experimental model trained on data from Labrador Retrievers (pure and Golden Retriever crosses) on dogs from another breed (Golden Retriever). A summary of the classification metrics on the golden set for Classifiers 1, 2, and 3 per class, and f1-weighted and f1-macro averages is shown in Tables 15–17, respectively. Classifier 3 again achieved the best f1-macro and f1-weighted metrics, it was the only model capable of correctly classifying body shakes.

**Table 15:** Classification metrics per posture achieved using the best models selected by grid search in Classifier 1 on the golden set.

Posture	TPR	TNR	Accuracy	PPV	F1-score
Body shake	0.00	1.00	1.00	0.00	0.00
Lying down	0.99	1.00	1.00	0.99	0.99
Sitting	0.97	0.99	0.98	0.91	0.94
Standing	0.96	0.92	0.94	0.91	0.93
Walking	0.87	0.98	0.95	0.96	0.91
f1-weighted	0.93				
f1-macro	0.75				

**Table 16:** Classification metrics per posture achieved using the best models selected by grid search in Classifier 2 on the golden set.

Posture	TPR	TNR	Accuracy	PPV	F1-score
Body shake	0.00	1.00	1.00	0.00	0.00
Lying down	0.99	1.00	1.00	1.00	1.00
Sitting	0.97	1.00	1.00	1.00	0.99
Standing	0.98	0.92	0.95	0.91	0.94
Walking	0.87	0.99	0.95	0.97	0.92
f1-weighted	0.94				
f1-macro	0.76				

**Fig 9:**
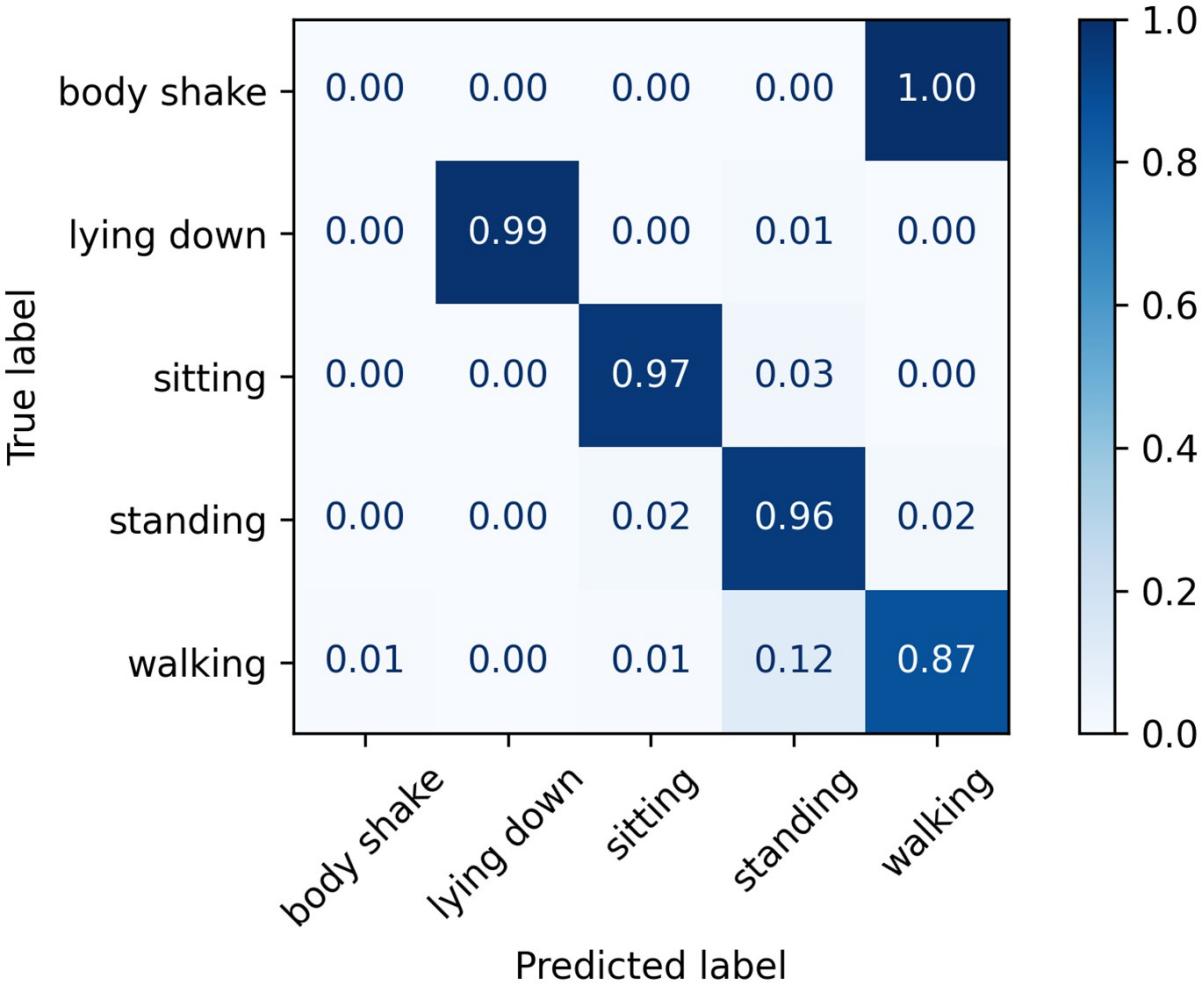
Confusion matrix for Classifier 1 on the golden set.

**Fig 10:**
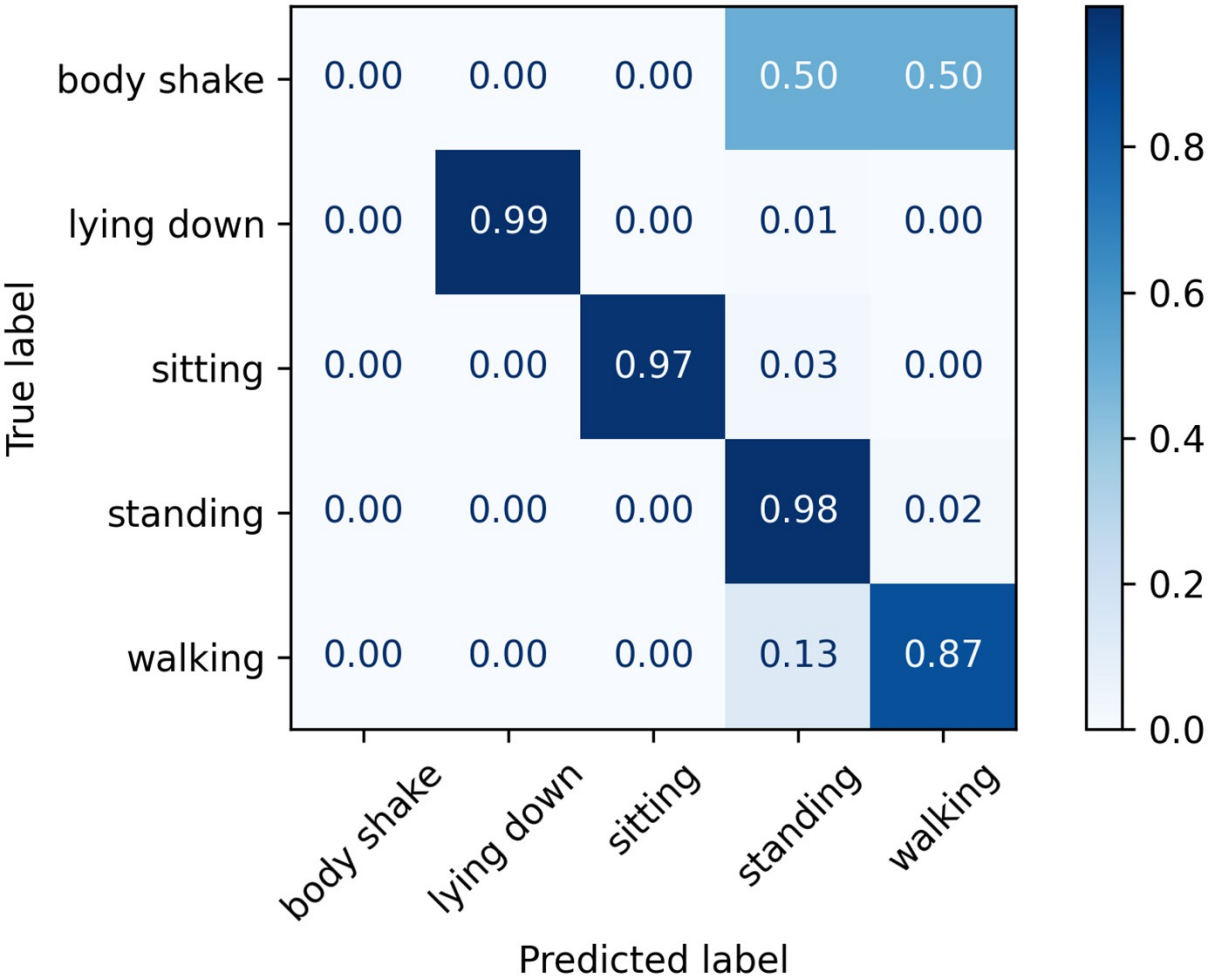
Confusion matrix for Classifier 2 on the golden set.

**Fig 11:**
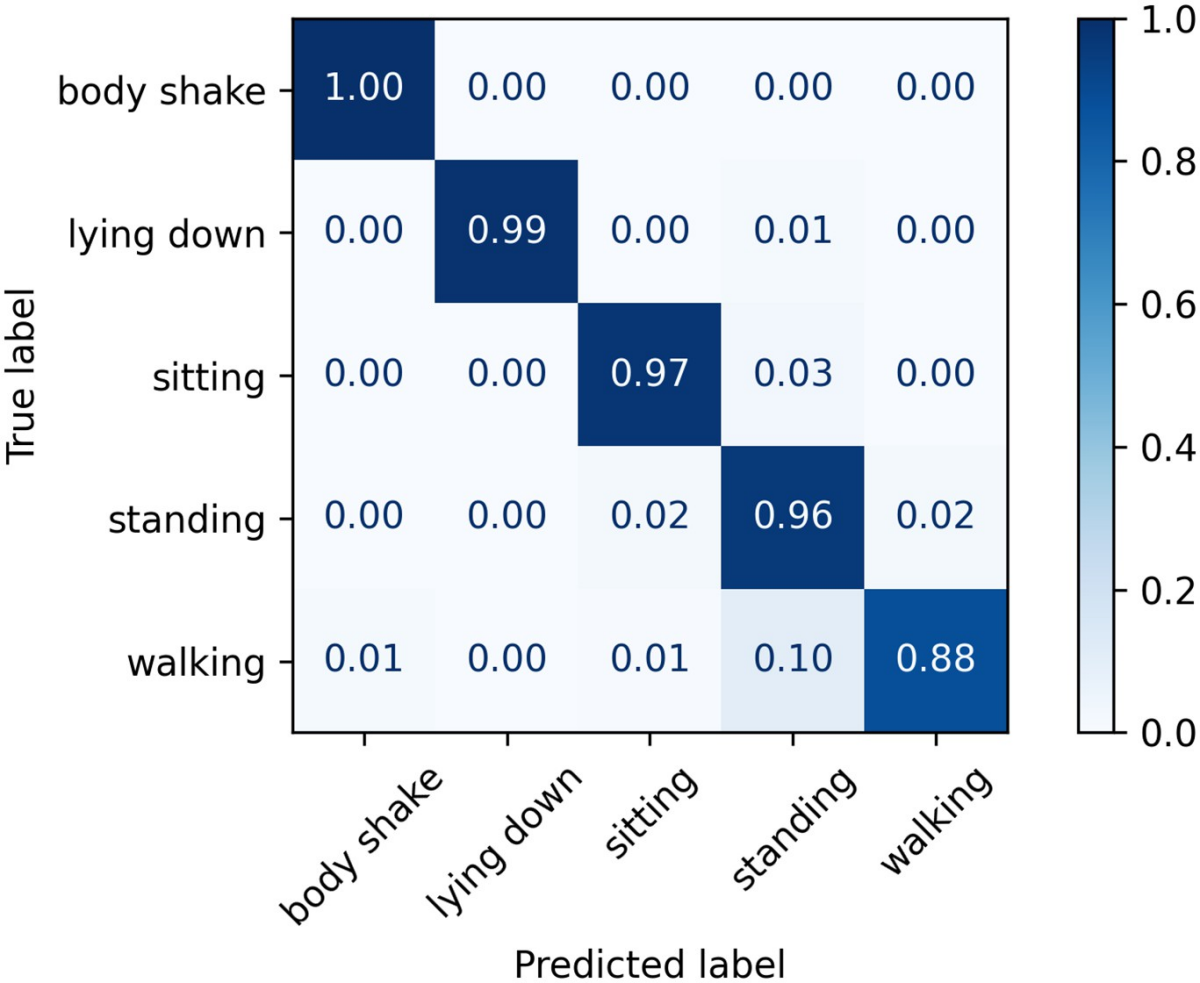
Confusion matrix for Classifier 3 on the golden set.

The normalised confusion matrices with the predicted labels for the golden set using Classifier 1, 2, and 3 models are shown in Figs 9–11, respectively. It can be visually seen that Classifier 3 achieves the overall best results in correctly classifying the classes.

**Table 17:** Classification metrics per posture achieved using the best models selected by grid search in Classifier 3 on the golden set.

Posture	TPR	TNR	Accuracy	PPV	F1-score
Body shake	1.00	1.00	1.00	0.29	0.44
Lying down	0.99	1.00	1.00	0.99	0.99
Sitting	0.97	0.99	0.99	0.92	0.95
Standing	0.96	0.93	0.95	0.92	0.94
Walking	0.88	0.99	0.95	0.97	0.92
f1-weighted	0.94				
f1-macro	0.85				

### 3.5 Best classifier

Intra-breed and inter-breed evaluation revealed that there were no significant performance differences between Golden Retrievers, Labrador Retrievers, and their crosses. Classifier 3 was selected as the best classifier considering the results from Section 3.1. In order to obtain unified performance metrics for the best model, Classifier 3 was used to calculate performance on the combined test and golden sets.

**Fig 12:**
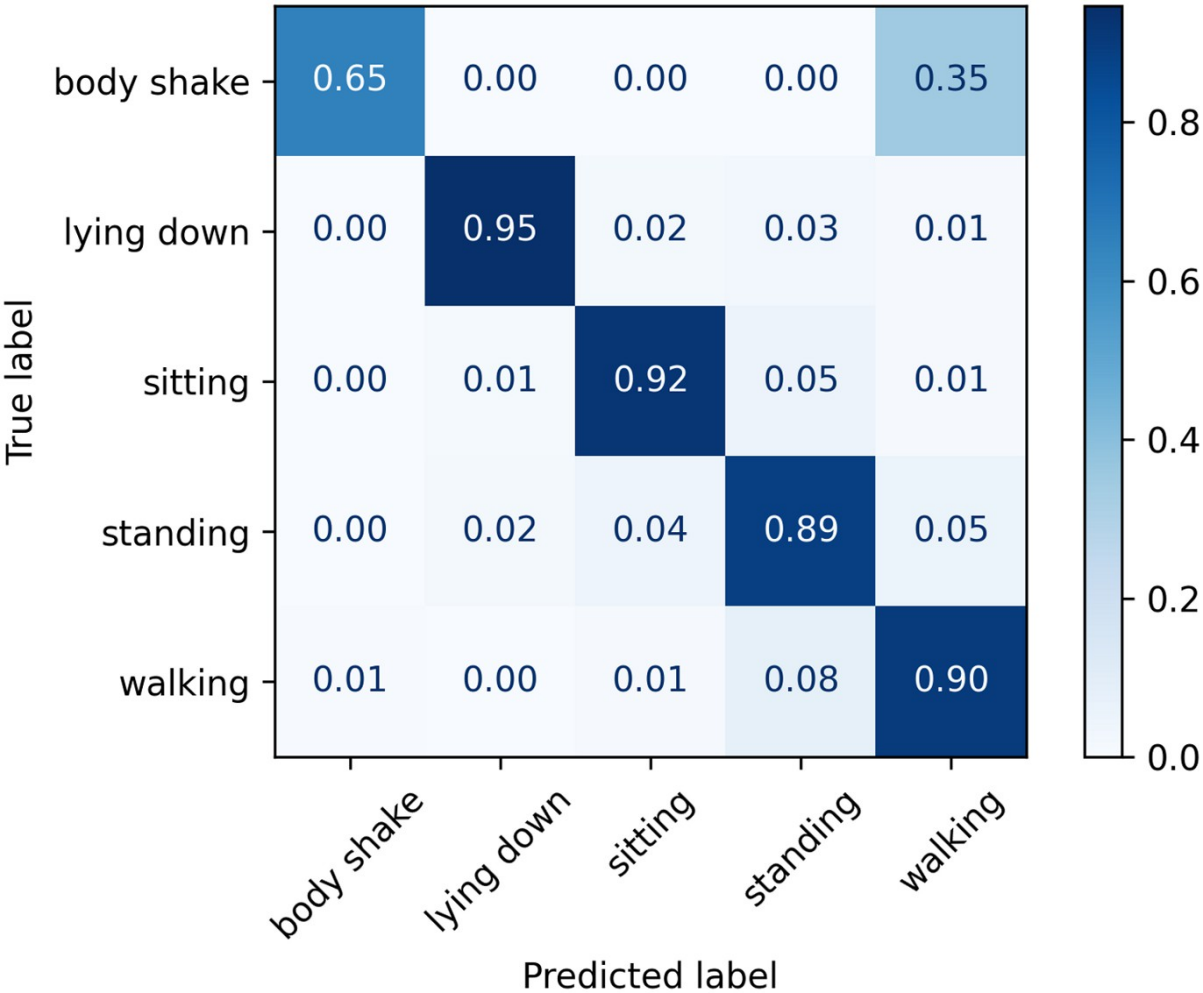
Confusion matrix for Classifier 3 on the test and golden sets.

A summary of the key classification metrics per class, and f1-weighted and f1-macro averages is shown in Table 18; and a confusion matrix is shown in Fig 12. These are considered the final results of the present work.

**Table 18:** Classification metrics per posture achieved using the best models selected by grid search in Classifier 3 on the test and golden sets combined.

Posture	TPR	TNR	Accuracy	PPV	F1-score
Body shake	0.65	1.00	1.00	0.45	0.53
Lying down	0.95	0.99	0.99	0.90	0.92
Sitting	0.92	0.97	0.96	0.88	0.90
Standing	0.89	0.94	0.91	0.92	0.90
Walking	0.90	0.96	0.95	0.91	0.91
f1-weighted	0.90				
f1-macro	0.83				

## 4 Discussion

The advantages of using IMUs over camera systems in the field of behaviour monitoring have already been established [51]. In the present work, a novel posture estimation system was developed specifically for working dogs. Accordingly, the data collection protocol designed includes popular working dog breeds and positions multiple IMUs on the harness. The main advantage of the posture estimation system developed in this work is the superior prediction performance achieved as demonstrated through comparison with previous research. The novel machine learning algorithm developed contributes to the state-of-the-art by employing advanced feature extraction techniques and experimenting with three different cascade architectures including anomaly detection models for the first time.

This was the first time that advanced feature extraction methods were applied to a canine posture recognition system. Feature extraction was performed with the Python package tsfel [49] using a rolling window, resulting in a dataset composed of 3,300 features and 30,224 observations. Features were chosen by the Select K Best algorithm based on the scores given by f-classification considering the label posture. The analysis of the collective contributions of the features deriving from each of the three IMUs placed on the dog’s neck, chest, and back revealed the importance of the back and chest sensors. Moreover, only features from the back and chest IMUs were selected by the algorithm, indicating that these are more informative of the targeted postures than the ones derived from the neck sensor. Brugarolas et al. [5] collected data from IMUs placed in similar positions on 7 dogs; however, only 2 of these dogs had IMUs placed on their chest and back sensors. Even though data were very limited, they suggested the significance of the rump, neck, and chest sensors which was confirmed in this research with a much bigger sample size (N = 42). Accelerometer data were more indicative of posture than gyroscope data as shown in Tables 9 and 10. This finding is in line with previous research [5, 7]. It is important to note that these results are influenced by the types of posture targeted in each study.

A grid search was deployed to find the optimal combination of hyper-parameters for the feature selection and classifier as outlined in Section 2.7. In particular, it used a 10-fold group cross-validation technique to calculate the performance in 10 different subsets of the development set while controlling for dogs to select the hyper-parameters that resulted in the best f1-weighted performance. The main drawback of the grid search algorithm is that it can lead to overfitting as it selects the model that delivers the best mean fold performance and does not take into account the gap between training and test performance or the model complexity.

Three classifier architectures were evaluated in order to improve the overall performance in correctly identifying postures despite the naturally unequal distribution of observations per label. Modifying the classifier architecture resulted in an improvement of the f1-score of the minority class (body shake) while maintaining a comparable performance on the majority classes (lying down, sitting, standing, and walking). This can be particularly observed from Classifier 1 and 3 model results in Tables 12 and 14 on the test set, and Tables 15 and 17 on the golden set. In the latter case, Classifiers 1 and 2 did not appropriately identify the body shake at all, while Classifier 3 correctly labelled all body shakes. In terms of training and validation times, Classifier 1 was the fastest, while Classifiers 2 and 3 took a similar amount of time.

One unexpected result was the superior performance of all classifiers on the golden set as compared to the test set. The only class performing less well was body shake as indicated by the f1-scores in Tables 12–14 on the test; and Tables 15–17 on the golden set. Interestingly, Gerencser et al. [7] also reported a similar result as the inter-breed model achieved higher performance than the intra-breed, from 70.3% to 73.6%, and 72.6% to 73.5% using only Malinois and Labradors data for training, respectively. This indicates that the variability deriving from the breed difference was less significant than the methodological limitations of the data collection and annotation procedures. Hence, this result suggests that this model can be successfully utilised on not only Labrador Retrievers and crosses with Golden Retrievers but also pure Golden Retrievers as well.

### 4.1 Comparison with previous work

Classifier 3 was chosen as the best classification model in the present work, and its performance on the test and golden sets combined as shown in Section 3.5 was compared to previous studies in Table 19. The inclusion criteria for such comparison considered only papers that (1) estimated canine posture using IMUs; (2) provided classification metrics per posture (body shake, lying down, sitting, standing, and walking); (3) evaluated performance on different dogs due to significant performance difference between intra-subject and inter-subject metrics [5, 7]. Considering the nine research papers in Table 1, five papers were excluded because they did not meet the criteria [5, 6, 8, 9, 12]. However, studies that utilised commercial systems were included [35, 39, 52].

**Table 19:** Comparison between Classifier 3 performance and previous studies reporting inter-subject classification performance metrics per posture. The best geometric means between TPR and TNR (g-mean) are in bold.

Publication	Posture	TPR	TNR	PPV	F1-score	G-mean
This study	Body shake	0.65	1.00	0.45	0.53	0.81
	Lying down	0.95	0.99	0.90	0.92	**0.97**
	Sitting	0.92	0.97	0.88	0.90	**0.95**
	Standing	0.89	0.94	0.92	0.90	**0.91**
	Walking	0.90	0.96	0.91	0.91	0.93
[39]	Body shake	0.916	1.000	0.795	0.851	**0.957**
	Lying down	0.826	0.913	0.724	0.772	0.868
	Sitting	0.409	0.915	0.347	0.375	0.612
	Standing	0.793	0.900	0.916	0.850	0.845
	Walking	0.903	0.969	0.706	0.792	0.935
[38]	Lying down	0.837				
	Sitting	0.899				
	Standing	0.941				
	Walking	0.945				
[35]	Walking	0.91	0.91	0.99	0.95	0.91
[52]	Walking	0.95	0.96	0.83	0.89	**0.95**
[7]	Lying down	0.722				
	Sitting	0.870				
	Standing	0.767				
	Walking	0.975				
[11]	Lying Down	0.68				
	Sitting	0.92				
	Standing	1.00				
	Walking	0.36				

Chambers et al. [39] recommended the use of sensitivity (TPR) and specificity (TNR) to compare the performance of classifiers trained on different datasets in order to control for class imbalance effects. Hence, both metrics were combined in a geometric mean (g-mean) for ease of comparison. The highest g-mean value for each posture was highlighted in bold.

Classifier 3 achieved the highest performance for the postures lying down, sitting, and standing on the combined test and golden sets. The best g-means for body shake and walking were reported by Chambers et al. [39] and Den Uijl et al. [52], respectively. Gerencser et al. [7] achieved the highest TPR for walking; however, there was insufficient information to calculate other performance metrics. It is important to note that, although Ribeiro et al. [11] reported a high TPR for sitting and standing, there were just a few observations from 5 dogs. Moreover, it was not clear whether the algorithm was built using data from the same dogs that were used in the test set.

Classifier 3 achieved the top overall performance on the five postures considering that it attained an arithmetic mean of the f1-score, also known as f1-macro, of 0.83 compared to 0.73 in Chambers et al. [39]. Furthermore, Classifier 3 produced an average TPR of 0.92 compared to 0.905, 0.83, 0.74 in Kumpulainen et al. [38], Gerencser et al. [7], and Ribeiro et al. [11], respectively, taking into account the postures lying down, sitting, standing, and walking. Classifier 3 has outperformed previous models published in the literature for predicting the five postures on dogs that did not belong to the original training set. Conclusively, these results indicate that the proposed system can outperform the state-of-the-art in a real environment.

### 4.2 Limitations and opportunities

As discussed, inaccurate annotations could account for a significant number of errors. Two main methodological limitations were identified to affect the precision of labels. Firstly, data synchronisation was done by showing the standard time on camera at the beginning of the video recording while this could be improved by shaking the sensor in front of the camera. Secondly, data annotation was done second by second, while it could be improved by using some video annotation software to increase the precision of the timestamps. These limitations can result in labels being misaligned with the data.

Other two causes of incorrect labels were identified: undefined transitions between two activities and rapid changes from one activity to another. The former case refers to the variability in the duration of transitions [11], for instance transitioning from walking to standing is nearly instantaneous; however, transitioning from standing to lying down can take a couple of seconds; such an observation could understandably be classified as sitting. The latter is observed in cases where the dog is walking but stands briefly only to resume walking again, such an observation could acceptably be classified as standing. This could explain the misclassification between such classes.

In order to address the first issue, a fixed transition time between postures was used when extracting features; however, it could still not have been long enough for short postures. The second issue is harder to address as sometimes the posture itself may not be very well defined. It is advisable to utilise video commenting software for data labelling, allowing higher precision in the annotations.

The minority class body shake was sometimes incorrectly classified as walking. Such results can be attributed to two main causes. Firstly, it is reasonable to estimate that a significant number of miss-classification cases could have resulted from inaccurate labels. This is because body shakes are very rapid movements typically happening between walking or standing postures. As annotations were made second by second, it is possible that some frames contain mixed postures. Secondly, these are the only dynamic postures while all others are static. It can be assumed that there exists a higher level of similarity between these postures when compared to all other postures, making it harder to distinguish them.

Different window sizes could be employed as some classes like body shake are shorter in duration than others. Accordingly, other techniques could be explored including KNN with Dynamic Time Warping [12], and deep learning methods such as convolutional neural network (CNN) [53] and long short-term memory (LSTM) motifs [39].

### 4.3 Recommendations

We suggest that researchers use the framework employed in this research. In particular, they are requested to make their datasets publicly available, describe key methodological details and expand their performance reports. This will allow for easier comparison, reproducibility, and knowledge transfer for the advancement of this field.

The following details are crucial and should be reported: data collection materials (sensor type and sampling rate); methods (number of dogs, breeds, context, types of behaviours, duration, repetitions); data pre-processing (window size and overlap); feature extraction (calculation, hyper-parameters); dataset statistics (size of training, development, and validation/test sets); dataset splitting criteria (e.g. breed, subject, number of observations); dataset details (number of observations per behaviour); prediction algorithm (type, hyper-parameters); optimisation (hyper-parameters and search space); and evaluation (technique, hyper-parameters, metrics, training, and testing time).

Future research should also test the performance of new dog activity recognition models on previously published datasets whenever possible to benchmark results. Researchers are encouraged to validate commercial canine activity monitoring devices, in terms of their performance in quantifying activity levels and identifying typical behaviours.

## 5 Conclusions

The main goal of this study was to investigate whether the present system comprising neck, back, and chest sensors would surpass the performance reported in published work on canine posture classification systems, namely: body shake, lying down, sitting, standing, and walking. This work contributes to the state-of-the-art knowledge in using IMUs for posture prediction using machine learning, by expanding the current understanding of sensor position, feature extraction, and importance as well as model architecture.

The importance of adding IMU sensors to the back and chest for more accurate posture prediction was demonstrated, encouraging the inclusion of IMU sensors on the dog harness for applications where the accuracy of posture predictions is critical, as in the case of some types of working dogs. Advanced feature extraction techniques for time series were been successfully employed and validated for the first time in the field of canine posture prediction. Feature selection was necessary to remove uninformative features, reducing the complexity of models and processing time.

The best-performing model architecture was Classifier 3 which comprised an anomaly detection estimator to identify the minority class (body shake) followed by a classifier to detect the other four postures (lying down, sitting, standing, and walking). The noticeably higher performance achieved by this novel classifier proves the advantage of combining different classifiers to leverage their respective strengths. In particular, this result indicates that the cascade machine learning architecture including the anomaly detection model significantly improved the detection rate of less frequent behaviour (body shake).

The novel canine posture estimator presented here achieved the best overall performance for predicting the five postures compared to previous research. The best performing canine posture estimator previously reported in the literature was presented in Chambers et al. [39]. This novel estimator attained a superior performance in terms of both f1-macro—0.83 versus 0.73—and mean g-mean—0.91 versus 0.84, respectively. Hence, this novel posture estimator system for working dogs provides the most correct predictions of five canine behaviours.

Finally, the IMU Posture dataset built using 42 working dogs was made publicly available. This allows the application of other machine learning techniques and a fair performance comparison between different methods. In particular, future research should investigate the use of other advanced feature selection techniques [54, 55], improve the explainability of models using techniques such as local interpretable model agnostic (LIME) and ELI5 [56, 57], and deep learning techniques (CNNs and LSTM networks) for the prediction of postures. Additional data should be added to the dataset to include different postures and other working dog breeds to create more complete automatic ethograms. These would enable the development of a range of applications to assist working dog organisations during dogs’ training and working lives.
